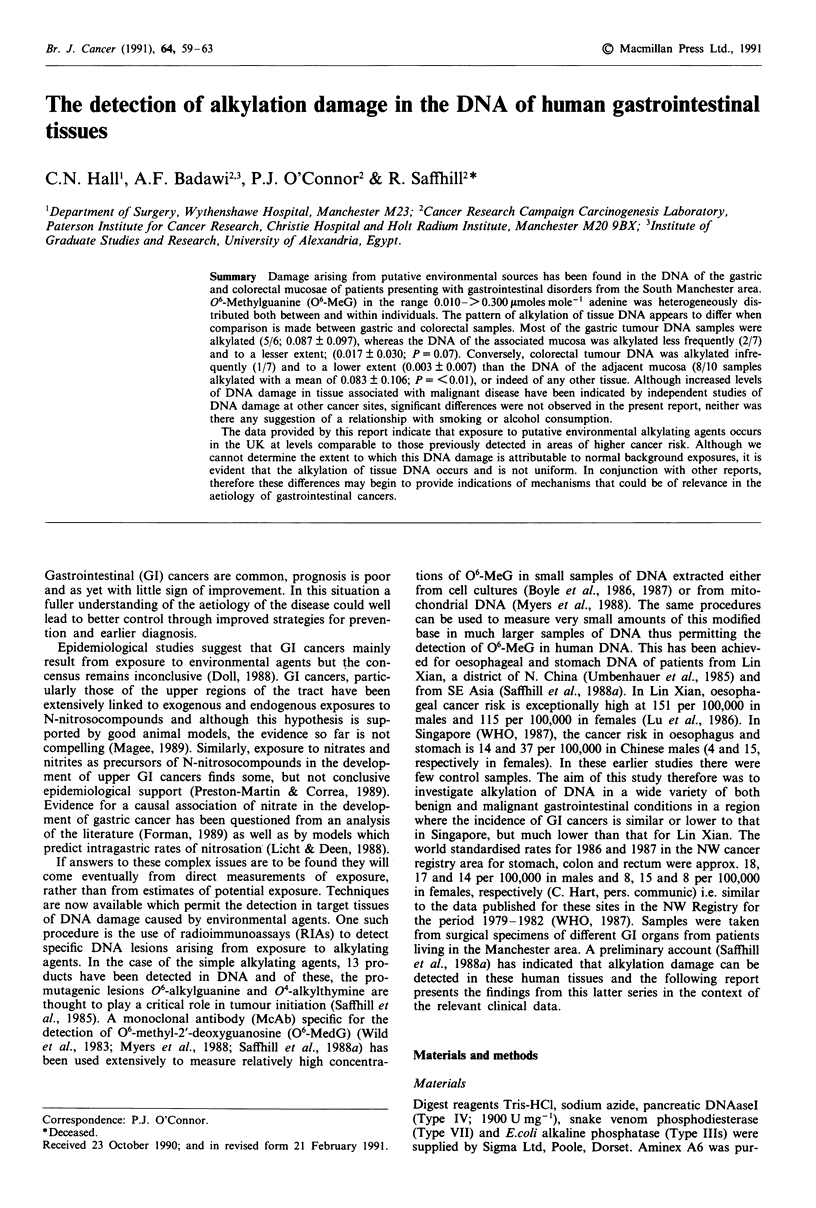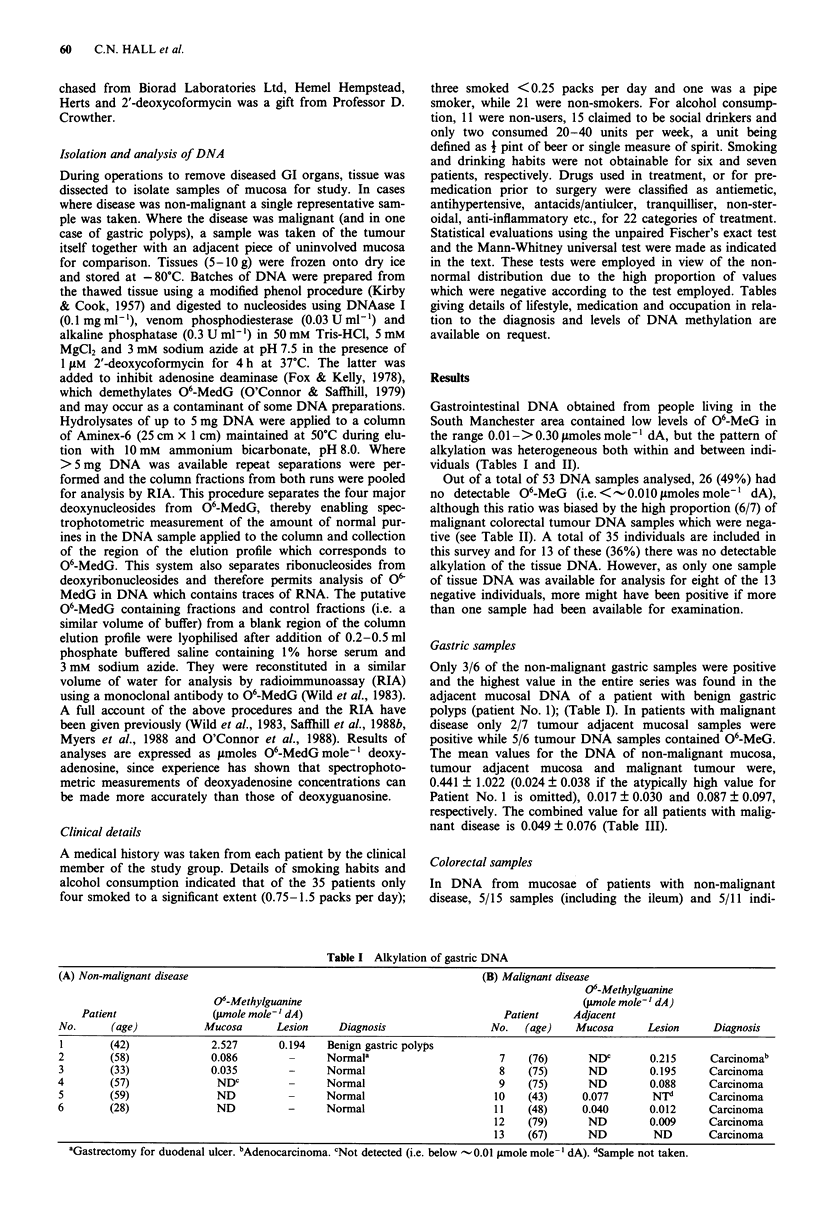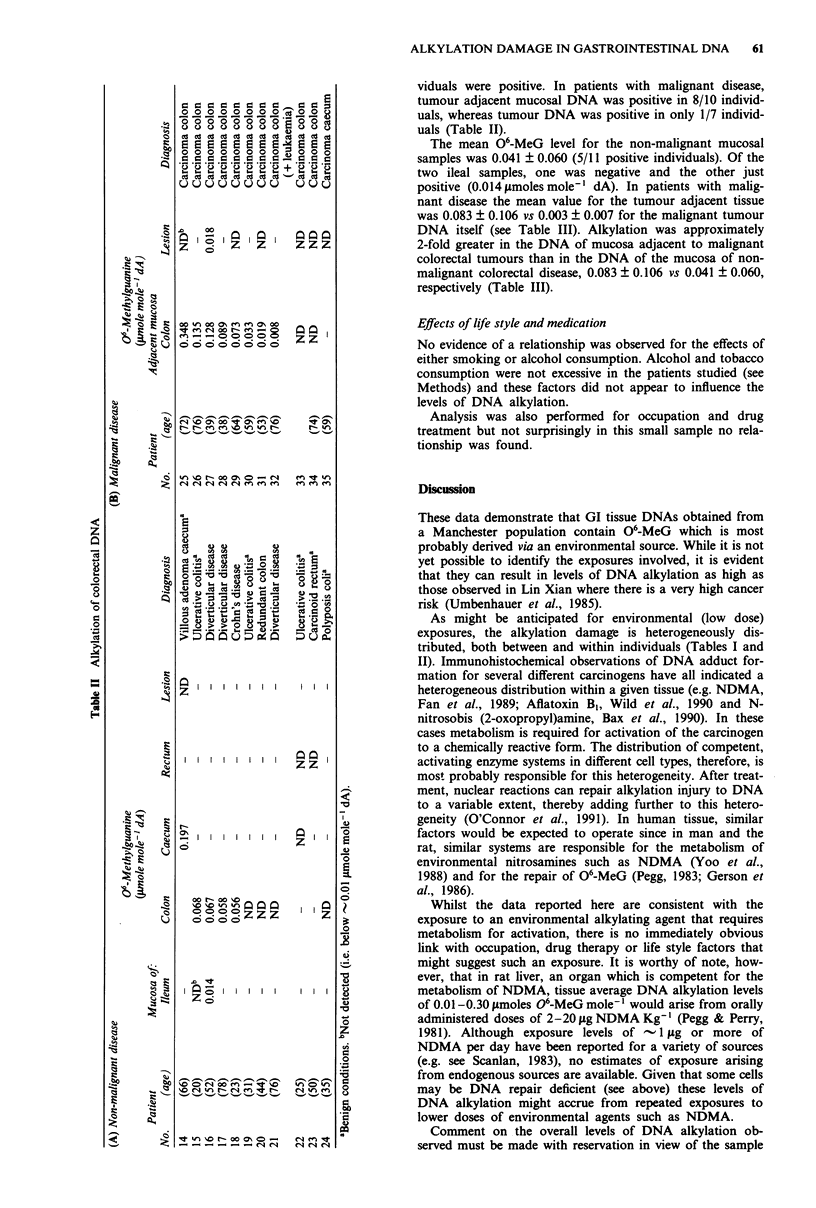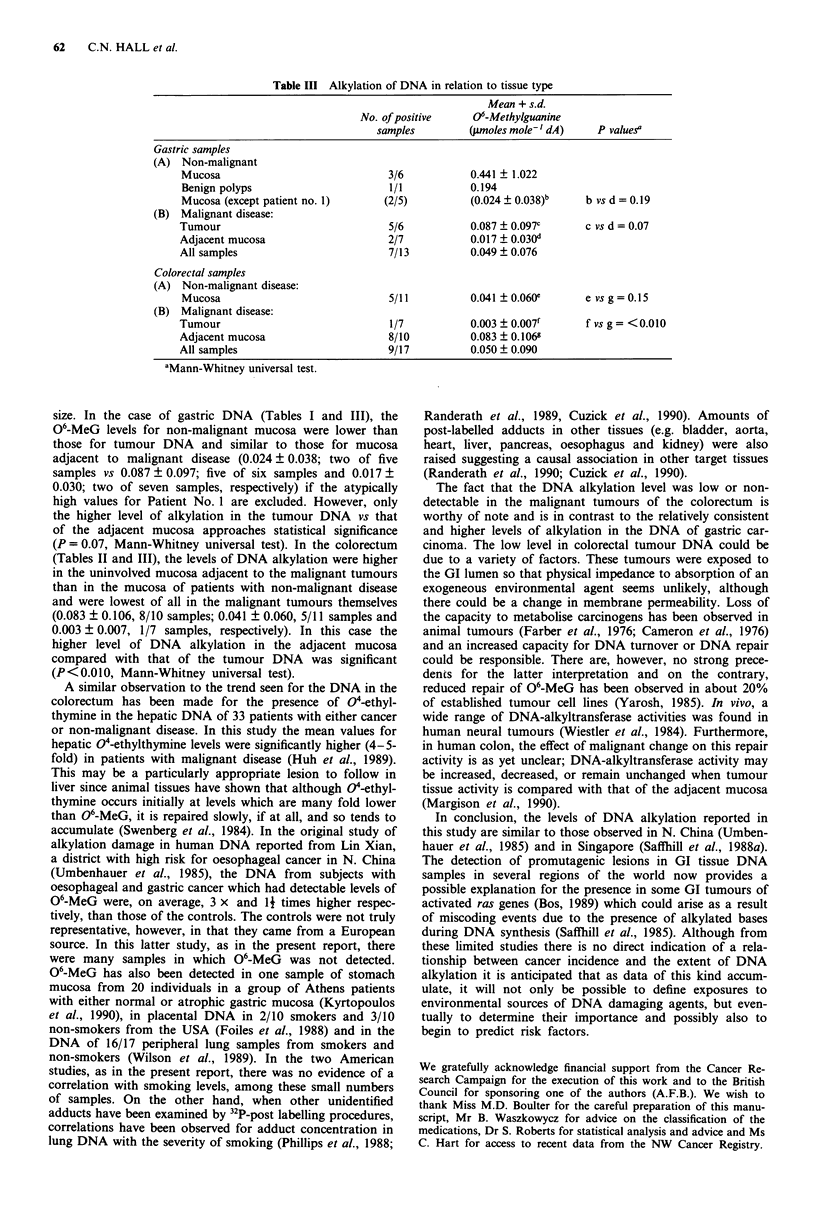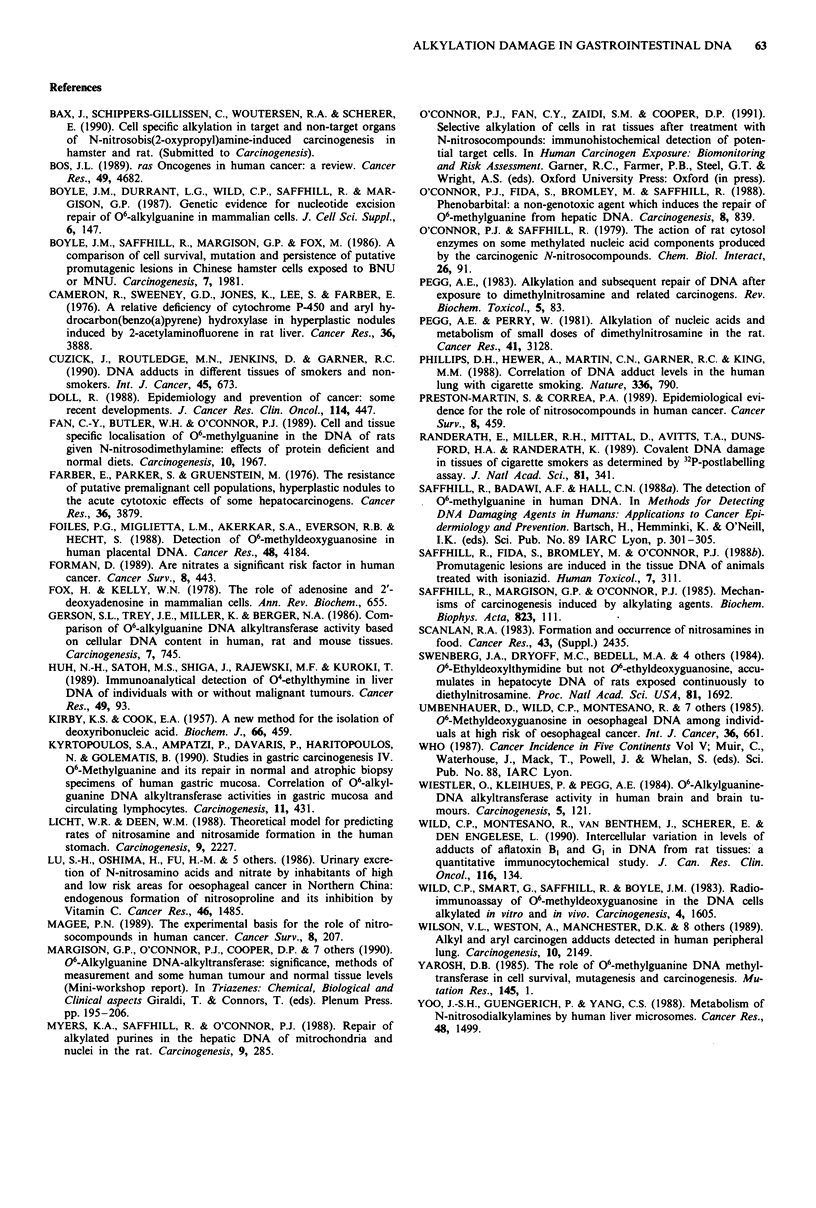# The detection of alkylation damage in the DNA of human gastrointestinal tissues.

**DOI:** 10.1038/bjc.1991.239

**Published:** 1991-07

**Authors:** C. N. Hall, A. F. Badawi, P. J. O'Connor, R. Saffhill

**Affiliations:** Department of Surgery, Wythenshawe Hospital, Manchester.

## Abstract

Damage arising from putative environmental sources has been found in the DNA of the gastric and colorectal mucosae of patients presenting with gastrointestinal disorders from the South Manchester area. O6-Methylguanine (O6-MeG) in the range 0.010- greater than 0.300 mu moles mole-1 adenine was heterogeneously distributed both between and within individuals. The pattern of alkylation of tissue DNA appears to differ when comparison is made between gastric and colorectal samples. Most of the gastric tumour DNA samples were alkylated (5/6; 0.087 +/- 0.097), whereas the DNA of the associated mucosa was alkylated less frequently (2/7) and to a lesser extent; (0.017 +/- 0.030; P = 0.07). Conversely, colorectal tumour DNA was alkylated infrequently (1/7) and to a lower extent (0.003 +/- 0.007) than the DNA of the adjacent mucosa (8/10 samples alkylated with a mean of 0.083 +/- 0.106; P = less than 0.01), or indeed of any other tissue. Although increased levels of DNA damage in tissue associated with malignant disease have been indicated by independent studies of DNA damage at other cancer sites, significant differences were not observed in the present report, neither was there any suggestion of a relationship with smoking or alcohol consumption. The data provided by this report indicate that exposure to putative environmental alkylating agents occurs in the UK at levels comparable to those previously detected in areas of higher cancer risk. Although we cannot determine the extent to which this DNA damage is attributable to normal background exposures, it is evident that the alkylation of tissue DNA occurs and is not uniform. In conjunction with other reports, therefore these differences may begin to provide indications of mechanisms that could be of relevance in the aetiology of gastrointestinal cancers.


Br. J. Cancer (1991), 64, 59–63 © Macmillan Press Ltd., 1991

### The detection of alkylation damage in the DNA of human gastrointestinal tissues

C.N. Hall[1], A.F. Badawi[2,3], P.J. O'Connor[2] & R. Saffhill[2]*

[1]*Department of Surgery, Wythenshawe Hospital, Manchester M23;* [2]*Cancer Research Campaign Carcinogenesis Laboratory, Paterson Institute for Cancer Research, Christie Hospital and Holt Radium Institute, Manchester M20 9BX;* [3]*Institute of Graduate Studies and Research, University of Alexandria, Egypt.*

**Summary** Damage arising from putative environmental sources has been found in the DNA of the gastric and colorectal mucosae of patients presenting with gastrointestinal disorders from the South Manchester area. $O^6$-Methylguanine ($O^6$-MeG) in the range 0.010–>0.300 μmoles $mole^{-1}$ adenine was heterogeneously distributed both between and within individuals. The pattern of alkylation of tissue DNA appears to differ when comparison is made between gastric and colorectal samples. Most of the gastric tumour DNA samples were alkylated (5/6; 0.087 ± 0.097), whereas the DNA of the associated mucosa was alkylated less frequently (2/7) and to a lesser extent; (0.017 ± 0.030; $P = 0.07$). Conversely, colorectal tumour DNA was alkylated infrequently (1/7) and to a lower extent (0.003 ± 0.007) than the DNA of the adjacent mucosa (8/10 samples alkylated with a mean of 0.083 ± 0.106; $P = <0.01$), or indeed of any other tissue. Although increased levels of DNA damage in tissue associated with malignant disease have been indicated by independent studies of DNA damage at other cancer sites, significant differences were not observed in the present report, neither was there any suggestion of a relationship with smoking or alcohol consumption.

The data provided by this report indicate that exposure to putative environmental alkylating agents occurs in the UK at levels comparable to those previously detected in areas of higher cancer risk. Although we cannot determine the extent to which this DNA damage is attributable to normal background exposures, it is evident that the alkylation of tissue DNA occurs and is not uniform. In conjunction with other reports, therefore these differences may begin to provide indications of mechanisms that could be of relevance in the aetiology of gastrointestinal cancers.

Gastrointestinal (GI) cancers are common, prognosis is poor and as yet with little sign of improvement. In this situation a fuller understanding of the aetiology of the disease could well lead to better control through improved strategies for prevention and earlier diagnosis.

Epidemiological studies suggest that GI cancers mainly result from exposure to environmental agents but the consensus remains inconclusive (Doll, 1988). GI cancers, particularly those of the upper regions of the tract have been extensively linked to exogenous and endogenous exposures to N-nitrosocompounds and although this hypothesis is supported by good animal models, the evidence so far is not compelling (Magee, 1989). Similarly, exposure to nitrates and nitrites as precursors of N-nitrosocompounds in the development of upper GI cancers finds some, but not conclusive epidemiological support (Preston-Martin & Correa, 1989). Evidence for a causal association of nitrate in the development of gastric cancer has been questioned from an analysis of the literature (Forman, 1989) as well as by models which predict intragastric rates of nitrosation (Licht & Deen, 1988).

If answers to these complex issues are to be found they will come eventually from direct measurements of exposure, rather than from estimates of potential exposure. Techniques are now available which permit the detection in target tissues of DNA damage caused by environmental agents. One such procedure is the use of radioimmunoassays (RIAs) to detect specific DNA lesions arising from exposure to alkylating agents. In the case of the simple alkylating agents, 13 products have been detected in DNA and of these, the promutagenic lesions $O^6$-alkylguanine and $O^4$-alkylthymine are thought to play a critical role in tumour initiation (Saffhill *et al.*, 1985). A monoclonal antibody (McAb) specific for the detection of $O^6$-methyl-2′-deoxyguanosine ($O^6$-MedG) (Wild *et al.*, 1983; Myers *et al.*, 1988; Saffhill *et al.*, 1988*a*) has been used extensively to measure relatively high concentrations of $O^6$-MeG in small samples of DNA extracted either from cell cultures (Boyle *et al.*, 1986, 1987) or from mitochondrial DNA (Myers *et al.*, 1988). The same procedures can be used to measure very small amounts of this modified base in much larger samples of DNA thus permitting the detection of $O^6$-MeG in human DNA. This has been achieved for oesophageal and stomach DNA of patients from Lin Xian, a district of N. China (Umbenhauer *et al.*, 1985) and from SE Asia (Saffhill *et al.*, 1988*a*). In Lin Xian, oesophageal cancer risk is exceptionally high at 151 per 100,000 in males and 115 per 100,000 in females (Lu *et al.*, 1986). In Singapore (WHO, 1987), the cancer risk in oesophagus and stomach is 14 and 37 per 100,000 in Chinese males (4 and 15, respectively in females). In these earlier studies there were few control samples. The aim of this study therefore was to investigate alkylation of DNA in a wide variety of both benign and malignant gastrointestinal conditions in a region where the incidence of GI cancers is similar or lower to that in Singapore, but much lower than that for Lin Xian. The world standardised rates for 1986 and 1987 in the NW cancer registry area for stomach, colon and rectum were approx. 18, 17 and 14 per 100,000 in males and 8, 15 and 8 per 100,000 in females, respectively (C. Hart, pers. communic) i.e. similar to the data published for these sites in the NW Registry for the period 1979–1982 (WHO, 1987). Samples were taken from surgical specimens of different GI organs from patients living in the Manchester area. A preliminary account (Saffhill *et al.*, 1988*a*) has indicated that alkylation damage can be detected in these human tissues and the following report presents the findings from this latter series in the context of the relevant clinical data.

### Materials and methods

### *Materials*

Digest reagents Tris-HCl, sodium azide, pancreatic DNAaseI (Type IV; 1900 U $mg^{-1}$), snake venom phosphodiesterase (Type VII) and *E.coli* alkaline phosphatase (Type IIIs) were supplied by Sigma Ltd, Poole, Dorset. Aminex A6 was pur-

Correspondence: P.J. O'Connor.
*Deceased.
Received 23 October 1990; and in revised form 21 February 1991.

chased from Biorad Laboratories Ltd, Hemel Hempstead, Herts and 2′-deoxycoformycin was a gift from Professor D. Crowther.

*Isolation and analysis of DNA*

During operations to remove diseased GI organs, tissue was dissected to isolate samples of mucosa for study. In cases where disease was non-malignant a single representative sample was taken. Where the disease was malignant (and in one case of gastric polyps), a sample was taken of the tumour itself together with an adjacent piece of uninvolved mucosa for comparison. Tissues (5–10 g) were frozen onto dry ice and stored at −80°C. Batches of DNA were prepared from the thawed tissue using a modified phenol procedure (Kirby & Cook, 1957) and digested to nucleosides using DNAase I (0.1 mg $ml^{-1}$), venom phosphodiesterase (0.03 U $ml^{-1}$) and alkaline phosphatase (0.3 U $ml^{-1}$) in 50 mM Tris-HCl, 5 mM $MgCl_2$ and 3 mM sodium azide at pH 7.5 in the presence of 1 μM 2′-deoxycoformycin for 4 h at 37°C. The latter was added to inhibit adenosine deaminase (Fox & Kelly, 1978), which demethylates $O^6$-MedG (O'Connor & Saffhill, 1979) and may occur as a contaminant of some DNA preparations. Hydrolysates of up to 5 mg DNA were applied to a column of Aminex-6 (25 cm × 1 cm) maintained at 50°C during elution with 10 mM ammonium bicarbonate, pH 8.0. Where >5 mg DNA was available repeat separations were performed and the column fractions from both runs were pooled for analysis by RIA. This procedure separates the four major deoxynucleosides from $O^6$-MedG, thereby enabling spectrophotometric measurement of the amount of normal purines in the DNA sample applied to the column and collection of the region of the elution profile which corresponds to $O^6$-MedG. This system also separates ribonucleosides from deoxyribonucleosides and therefore permits analysis of $O^6$-MedG in DNA which contains traces of RNA. The putative $O^6$-MedG containing fractions and control fractions (i.e. a similar volume of buffer) from a blank region of the column elution profile were lyophilised after addition of 0.2–0.5 ml phosphate buffered saline containing 1% horse serum and 3 mM sodium azide. They were reconstituted in a similar volume of water for analysis by radioimmunoassay (RIA) using a monoclonal antibody to $O^6$-MedG (Wild *et al.*, 1983). A full account of the above procedures and the RIA have been given previously (Wild *et al.*, 1983, Saffhill *et al.*, 1988*b*, Myers *et al.*, 1988 and O'Connor *et al.*, 1988). Results of analyses are expressed as μmoles $O^6$-MedG $mole^{-1}$ deoxyadenosine, since experience has shown that spectrophotometric measurements of deoxyadenosine concentrations can be made more accurately than those of deoxyguanosine.

*Clinical details*

A medical history was taken from each patient by the clinical member of the study group. Details of smoking habits and alcohol consumption indicated that of the 35 patients only four smoked to a significant extent (0.75–1.5 packs per day); three smoked <0.25 packs per day and one was a pipe smoker, while 21 were non-smokers. For alcohol consumption, 11 were non-users, 15 claimed to be social drinkers and only two consumed 20–40 units per week, a unit being defined as ½ pint of beer or single measure of spirit. Smoking and drinking habits were not obtainable for six and seven patients, respectively. Drugs used in treatment, or for premedication prior to surgery were classified as antiemetic, antihypertensive, antacids/antiulcer, tranquilliser, non-steroidal, anti-inflammatory etc., for 22 categories of treatment. Statistical evaluations using the unpaired Fischer's exact test and the Mann-Whitney universal test were made as indicated in the text. These tests were employed in view of the non-normal distribution due to the high proportion of values which were negative according to the test employed. Tables giving details of lifestyle, medication and occupation in relation to the diagnosis and levels of DNA methylation are available on request.

### Results

Gastrointestinal DNA obtained from people living in the South Manchester area contained low levels of $O^6$-MeG in the range 0.01–>0.30 μmoles $mole^{-1}$ dA, but the pattern of alkylation was heterogeneous both within and between individuals (Tables I and II).

Out of a total of 53 DNA samples analysed, 26 (49%) had no detectable $O^6$-MeG (i.e. <∼0.010 μmoles $mole^{-1}$ dA), although this ratio was biased by the high proportion (6/7) of malignant colorectal tumour DNA samples which were negative (see Table II). A total of 35 individuals are included in this survey and for 13 of these (36%) there was no detectable alkylation of the tissue DNA. However, as only one sample of tissue DNA was available for analysis for eight of the 13 negative individuals, more might have been positive if more than one sample had been available for examination.

*Gastric samples*

Only 3/6 of the non-malignant gastric samples were positive and the highest value in the entire series was found in the adjacent mucosal DNA of a patient with benign gastric polyps (patient No. 1); (Table I). In patients with malignant disease only 2/7 tumour adjacent mucosal samples were positive while 5/6 tumour DNA samples contained $O^6$-MeG. The mean values for the DNA of non-malignant mucosa, tumour adjacent mucosa and malignant tumour were, 0.441 ± 1.022 (0.024 ± 0.038 if the atypically high value for Patient No. 1 is omitted), 0.017 ± 0.030 and 0.087 ± 0.097, respectively. The combined value for all patients with malignant disease is 0.049 ± 0.076 (Table III).

*Colorectal samples*

In DNA from mucosae of patients with non-malignant disease, 5/15 samples (including the ileum) and 5/11 indi-

**Table I** Alkylation of gastric DNA

| (A) *Non-malignant disease* | | | | | (B) *Malignant disease* | | | | |
|---|---|---|---|---|---|---|---|---|---|
| *Patient* | | *$O^6$-Methylguanine (μmole $mole^{-1}$ dA)* | | | *Patient* | | *$O^6$-Methylguanine (μmole $mole^{-1}$ dA)* | | |
| *No.* | *(age)* | *Mucosa* | *Lesion* | *Diagnosis* | *No.* | *(age)* | *Adjacent Mucosa* | *Lesion* | *Diagnosis* |
| 1 | (42) | 2.527 | 0.194 | Benign gastric polyps | | | | | |
| 2 | (58) | 0.086 | – | Normal[a] | 7 | (76) | ND[c] | 0.215 | Carcinoma[b] |
| 3 | (33) | 0.035 | – | Normal | 8 | (75) | ND | 0.195 | Carcinoma |
| 4 | (57) | ND[c] | – | Normal | 9 | (75) | ND | 0.088 | Carcinoma |
| 5 | (59) | ND | – | Normal | 10 | (43) | 0.077 | NT[d] | Carcinoma |
| 6 | (28) | ND | – | Normal | 11 | (48) | 0.040 | 0.012 | Carcinoma |
| | | | | | 12 | (79) | ND | 0.009 | Carcinoma |
| | | | | | 13 | (67) | ND | ND | Carcinoma |

[a]Gastrectomy for duodenal ulcer. [b]Adenocarcinoma. [c]Not detected (i.e. below ∼0.01 μmole $mole^{-1}$ dA). [d]Sample not taken.

**Table II** Alkylation of colorectal DNA

(A) *Non-malignant disease*

| *Patient No.* | *(age)* | *Mucosa of: Ileum* | $O^6$-*Methylguanine (μmole mole$^{-1}$ dA)* *Colon* | *Caecum* | *Rectum* | *Lesion* | *Diagnosis* |
|---|---|---|---|---|---|---|---|
| 14 | (66) | – | | 0.197 | – | ND | Villous adenoma caecum[a] |
| 15 | (20) | ND[b] | 0.068 | – | – | – | Ulcerative colitis[a] |
| 16 | (52) | 0.014 | 0.067 | – | – | – | Diverticular disease |
| 17 | (78) | – | 0.058 | – | – | – | Diverticular disease |
| 18 | (23) | – | 0.056 | – | – | – | Crohn's disease |
| 19 | (31) | – | ND | – | – | – | Ulcerative colitis[a] |
| 20 | (44) | – | ND | – | – | – | Redundant colon |
| 21 | (76) | – | ND | – | – | – | Diverticular disease |
| 22 | (25) | – | – | ND | ND | – | Ulcerative colitis[a] |
| 23 | (50) | – | – | – | ND | – | Carcinoid rectum[a] |
| 24 | (35) | – | ND | – | – | – | Polyposis coli[a] |

(B) *Malignant disease*

| *Patient No.* | *(age)* | $O^6$-*Methylguanine (μmole mole$^{-1}$ dA)* *Adjacent mucosa Colon* | *Lesion* | *Diagnosis* |
|---|---|---|---|---|
| 25 | (72) | 0.348 | ND[b] | Carcinoma colon |
| 26 | (76) | 0.135 | – | Carcinoma colon |
| 27 | (39) | 0.128 | 0.018 | Carcinoma colon |
| 28 | (38) | 0.089 | – | Carcinoma colon |
| 29 | (64) | 0.073 | ND | Carcinoma colon |
| 30 | (59) | 0.033 | – | Carcinoma colon |
| 31 | (53) | 0.019 | ND | Carcinoma colon |
| 32 | (76) | 0.008 | – | Carcinoma colon (+ leukaemia) |
| 33 | | ND | ND | Carcinoma colon |
| 34 | (74) | ND | ND | Carcinoma colon |
| 35 | (59) | – | ND | Carcinoma caecum |

[a]Benign conditions. [b]Not detected (i.e. below ~0.01 μmole mole$^{-1}$ dA).

viduals were positive. In patients with malignant disease, tumour adjacent mucosal DNA was positive in 8/10 individuals, whereas tumour DNA was positive in only 1/7 individuals (Table II).

The mean $O^6$-MeG level for the non-malignant mucosal samples was 0.041 ± 0.060 (5/11 positive individuals). Of the two ileal samples, one was negative and the other just positive (0.014 μmoles mole$^{-1}$ dA). In patients with malignant disease the mean value for the tumour adjacent tissue was 0.083 ± 0.106 *vs* 0.003 ± 0.007 for the malignant tumour DNA itself (see Table III). Alkylation was approximately 2-fold greater in the DNA of mucosa adjacent to malignant colorectal tumours than in the DNA of the mucosa of non-malignant colorectal disease, 0.083 ± 0.106 *vs* 0.041 ± 0.060, respectively (Table III).

### *Effects of life style and medication*

No evidence of a relationship was observed for the effects of either smoking or alcohol consumption. Alcohol and tobacco consumption were not excessive in the patients studied (see Methods) and these factors did not appear to influence the levels of DNA alkylation.

Analysis was also performed for occupation and drug treatment but not surprisingly in this small sample no relationship was found.

### Discussion

These data demonstrate that GI tissue DNAs obtained from a Manchester population contain $O^6$-MeG which is most probably derived *via* an environmental source. While it is not yet possible to identify the exposures involved, it is evident that they can result in levels of DNA alkylation as high as those observed in Lin Xian where there is a very high cancer risk (Umbenhauer *et al.*, 1985).

As might be anticipated for environmental (low dose) exposures, the alkylation damage is heterogeneously distributed, both between and within individuals (Tables I and II). Immunohistochemical observations of DNA adduct formation for several different carcinogens have all indicated a heterogeneous distribution within a given tissue (e.g. NDMA, Fan *et al.*, 1989; Aflatoxin $B_1$, Wild *et al.*, 1990 and N-nitrosobis (2-oxopropyl)amine, Bax *et al.*, 1990). In these cases metabolism is required for activation of the carcinogen to a chemically reactive form. The distribution of competent, activating enzyme systems in different cell types, therefore, is most probably responsible for this heterogeneity. After treatment, nuclear reactions can repair alkylation injury to DNA to a variable extent, thereby adding further to this heterogeneity (O'Connor *et al.*, 1991). In human tissue, similar factors would be expected to operate since in man and the rat, similar systems are responsible for the metabolism of environmental nitrosamines such as NDMA (Yoo *et al.*, 1988) and for the repair of $O^6$-MeG (Pegg, 1983; Gerson *et al.*, 1986).

Whilst the data reported here are consistent with the exposure to an environmental alkylating agent that requires metabolism for activation, there is no immediately obvious link with occupation, drug therapy or life style factors that might suggest such an exposure. It is worthy of note, however, that in rat liver, an organ which is competent for the metabolism of NDMA, tissue average DNA alkylation levels of 0.01–0.30 μmoles $O^6$-MeG mole$^{-1}$ would arise from orally administered doses of 2–20 μg NDMA Kg$^{-1}$ (Pegg & Perry, 1981). Although exposure levels of ~1 μg or more of NDMA per day have been reported for a variety of sources (e.g. see Scanlan, 1983), no estimates of exposure arising from endogenous sources are available. Given that some cells may be DNA repair deficient (see above) these levels of DNA alkylation might accrue from repeated exposures to lower doses of environmental agents such as NDMA.

Comment on the overall levels of DNA alkylation observed must be made with reservation in view of the sample

**Table III** Alkylation of DNA in relation to tissue type

| | No. of positive samples | Mean + s.d. $O^6$-Methylguanine ($\mu$moles mole$^{-1}$ dA) | P values[a] |
|---|---|---|---|
| *Gastric samples* | | | |
| (A) Non-malignant | | | |
| Mucosa | 3/6 | 0.441 ± 1.022 | |
| Benign polyps | 1/1 | 0.194 | |
| Mucosa (except patient no. 1) | (2/5) | (0.024 ± 0.038)[b] | b *vs* d = 0.19 |
| (B) Malignant disease: | | | |
| Tumour | 5/6 | 0.087 ± 0.097[c] | c *vs* d = 0.07 |
| Adjacent mucosa | 2/7 | 0.017 ± 0.030[d] | |
| All samples | 7/13 | 0.049 ± 0.076 | |
| *Colorectal samples* | | | |
| (A) Non-malignant disease: | | | |
| Mucosa | 5/11 | 0.041 ± 0.060[e] | e *vs* g = 0.15 |
| (B) Malignant disease: | | | |
| Tumour | 1/7 | 0.003 ± 0.007[f] | f *vs* g = <0.010 |
| Adjacent mucosa | 8/10 | 0.083 ± 0.106[g] | |
| All samples | 9/17 | 0.050 ± 0.090 | |

[a]Mann-Whitney universal test.

size. In the case of gastric DNA (Tables I and III), the $O^6$-MeG levels for non-malignant mucosa were lower than those for tumour DNA and similar to those for mucosa adjacent to malignant disease (0.024 ± 0.038; two of five samples *vs* 0.087 ± 0.097; five of six samples and 0.017 ± 0.030; two of seven samples, respectively) if the atypically high values for Patient No. 1 are excluded. However, only the higher level of alkylation in the tumour DNA *vs* that of the adjacent mucosa approaches statistical significance ($P = 0.07$, Mann-Whitney universal test). In the colorectum (Tables II and III), the levels of DNA alkylation were higher in the uninvolved mucosa adjacent to the malignant tumours than in the mucosa of patients with non-malignant disease and were lowest of all in the malignant tumours themselves (0.083 ± 0.106, 8/10 samples; 0.041 ± 0.060, 5/11 samples and 0.003 ± 0.007, 1/7 samples, respectively). In this case the higher level of DNA alkylation in the adjacent mucosa compared with that of the tumour DNA was significant ($P < 0.010$, Mann-Whitney universal test).

A similar observation to the trend seen for the DNA in the colorectum has been made for the presence of $O^4$-ethylthymine in the hepatic DNA of 33 patients with either cancer or non-malignant disease. In this study the mean values for hepatic $O^4$-ethylthymine levels were significantly higher (4–5-fold) in patients with malignant disease (Huh *et al.*, 1989). This may be a particularly appropriate lesion to follow in liver since animal tissues have shown that although $O^4$-ethylthymine occurs initially at levels which are many fold lower than $O^6$-MeG, it is repaired slowly, if at all, and so tends to accumulate (Swenberg *et al.*, 1984). In the original study of alkylation damage in human DNA reported from Lin Xian, a district with high risk for oesophageal cancer in N. China (Umbenhauer *et al.*, 1985), the DNA from subjects with oesophageal and gastric cancer which had detectable levels of $O^6$-MeG were, on average, 3 × and 1½ times higher respectively, than those of the controls. The controls were not truly representative, however, in that they came from a European source. In this latter study, as in the present report, there were many samples in which $O^6$-MeG was not detected. $O^6$-MeG has also been detected in one sample of stomach mucosa from 20 individuals in a group of Athens patients with either normal or atrophic gastric mucosa (Kyrtopoulos *et al.*, 1990), in placental DNA in 2/10 smokers and 3/10 non-smokers from the USA (Foiles *et al.*, 1988) and in the DNA of 16/17 peripheral lung samples from smokers and non-smokers (Wilson *et al.*, 1989). In the two American studies, as in the present report, there was no evidence of a correlation with smoking levels, among these small numbers of samples. On the other hand, when other unidentified adducts have been examined by $^{32}$P-post labelling procedures, correlations have been observed for adduct concentration in lung DNA with the severity of smoking (Phillips *et al.*, 1988; Randerath *et al.*, 1989, Cuzick *et al.*, 1990). Amounts of post-labelled adducts in other tissues (e.g. bladder, aorta, heart, liver, pancreas, oesophagus and kidney) were also raised suggesting a causal association in other target tissues (Randerath *et al.*, 1990; Cuzick *et al.*, 1990).

The fact that the DNA alkylation level was low or non-detectable in the malignant tumours of the colorectum is worthy of note and is in contrast to the relatively consistent and higher levels of alkylation in the DNA of gastric carcinoma. The low level in colorectal tumour DNA could be due to a variety of factors. These tumours were exposed to the GI lumen so that physical impedance to absorption of an exogeneous environmental agent seems unlikely, although there could be a change in membrane permeability. Loss of the capacity to metabolise carcinogens has been observed in animal tumours (Farber *et al.*, 1976; Cameron *et al.*, 1976) and an increased capacity for DNA turnover or DNA repair could be responsible. There are, however, no strong precedents for the latter interpretation and on the contrary, reduced repair of $O^6$-MeG has been observed in about 20% of established tumour cell lines (Yarosh, 1985). *In vivo*, a wide range of DNA-alkyltransferase activities was found in human neural tumours (Wiestler *et al.*, 1984). Furthermore, in human colon, the effect of malignant change on this repair activity is as yet unclear; DNA-alkyltransferase activity may be increased, decreased, or remain unchanged when tumour tissue activity is compared with that of the adjacent mucosa (Margison *et al.*, 1990).

In conclusion, the levels of DNA alkylation reported in this study are similar to those observed in N. China (Umbenhauer *et al.*, 1985) and in Singapore (Saffhill *et al.*, 1988*a*). The detection of promutagenic lesions in GI tissue DNA samples in several regions of the world now provides a possible explanation for the presence in some GI tumours of activated *ras* genes (Bos, 1989) which could arise as a result of miscoding events due to the presence of alkylated bases during DNA synthesis (Saffhill *et al.*, 1985). Although from these limited studies there is no direct indication of a relationship between cancer incidence and the extent of DNA alkylation it is anticipated that as data of this kind accumulate, it will not only be possible to define exposures to environmental sources of DNA damaging agents, but eventually to determine their importance and possibly also to begin to predict risk factors.

We gratefully acknowledge financial support from the Cancer Research Campaign for the execution of this work and to the British Council for sponsoring one of the authors (A.F.B.). We wish to thank Miss M.D. Boulter for the careful preparation of this manuscript, Mr B. Waszkowycz for advice on the classification of the medications, Dr S. Roberts for statistical analysis and advice and Ms C. Hart for access to recent data from the NW Cancer Registry.

### References

BAX, J., SCHIPPERS-GILLISSEN, C., WOUTERSEN, R.A. & SCHERER, E. (1990). Cell specific alkylation in target and non-target organs of N-nitrosobis(2-oxypropyl)amine-induced carcinogenesis in hamster and rat. (Submitted to *Carcinogenesis*).

BOS, J.L. (1989). *ras* Oncogenes in human cancer: a review. *Cancer Res.*, **49**, 4682.

BOYLE, J.M., DURRANT, L.G., WILD, C.P., SAFFHILL, R. & MARGISON, G.P. (1987). Genetic evidence for nucleotide excision repair of $O^6$-alkylguanine in mammalian cells. *J. Cell Sci. Suppl.*, **6**, 147.

BOYLE, J.M., SAFFHILL, R., MARGISON, G.P. & FOX, M. (1986). A comparison of cell survival, mutation and persistence of putative promutagenic lesions in Chinese hamster cells exposed to BNU or MNU. *Carcinogenesis*, **7**, 1981.

CAMERON, R., SWEENEY, G.D., JONES, K., LEE, S. & FARBER, E. (1976). A relative deficiency of cytochrome P-450 and aryl hydrocarbon(benzo(a)pyrene) hydroxylase in hyperplastic nodules induced by 2-acetylaminofluorene in rat liver. *Cancer Res.*, **36**, 3888.

CUZICK, J., ROUTLEDGE, M.N., JENKINS, D. & GARNER, R.C. (1990). DNA adducts in different tissues of smokers and non-smokers. *Int. J. Cancer*, **45**, 673.

DOLL, R. (1988). Epidemiology and prevention of cancer: some recent developments. *J. Cancer Res. Clin. Oncol.*, **114**, 447.

FAN, C.-Y., BUTLER, W.H. & O'CONNOR, P.J. (1989). Cell and tissue specific localisation of $O^6$-methylguanine in the DNA of rats given N-nitrosodimethylamine: effects of protein deficient and normal diets. *Carcinogenesis*, **10**, 1967.

FARBER, E., PARKER, S. & GRUENSTEIN, M. (1976). The resistance of putative premalignant cell populations, hyperplastic nodules to the acute cytotoxic effects of some hepatocarcinogens. *Cancer Res.*, **36**, 3879.

FOILES, P.G., MIGLIETTA, L.M., AKERKAR, S.A., EVERSON, R.B. & HECHT, S. (1988). Detection of $O^6$-methyldeoxyguanosine in human placental DNA. *Cancer Res.*, **48**, 4184.

FORMAN, D. (1989). Are nitrates a significant risk factor in human cancer. *Cancer Surv.*, **8**, 443.

FOX, H. & KELLY, W.N. (1978). The role of adenosine and 2'-deoxyadenosine in mammalian cells. *Ann. Rev. Biochem.*, 655.

GERSON, S.L., TREY, J.E., MILLER, K. & BERGER, N.A. (1986). Comparison of $O^6$-alkylguanine DNA alkyltransferase activity based on cellular DNA content in human, rat and mouse tissues. *Carcinogenesis*, **7**, 745.

HUH, N.-H., SATOH, M.S., SHIGA, J., RAJEWSKI, M.F. & KUROKI, T. (1989). Immunoanalytical detection of $O^4$-ethylthymine in liver DNA of individuals with or without malignant tumours. *Cancer Res.*, **49**, 93.

KIRBY, K.S. & COOK, E.A. (1957). A new method for the isolation of deoxyribonucleic acid. *Biochem. J.*, **66**, 459.

KYRTOPOULOS, S.A., AMPATZI, P., DAVARIS, P., HARITOPOULOS, N. & GOLEMATIS, B. (1990). Studies in gastric carcinogenesis IV. $O^6$-Methylguanine and its repair in normal and atrophic biopsy specimens of human gastric mucosa. Correlation of $O^6$-alkylguanine DNA alkyltransferase activities in gastric mucosa and circulating lymphocytes. *Carcinogenesis*, **11**, 431.

LICHT, W.R. & DEEN, W.M. (1988). Theoretical model for predicting rates of nitrosamine and nitrosamide formation in the human stomach. *Carcinogenesis*, **9**, 2227.

LU, S.-H., OSHIMA, H., FU, H.-M. & 5 others. (1986). Urinary excretion of N-nitrosamino acids and nitrate by inhabitants of high and low risk areas for oesophageal cancer in Northern China: endogenous formation of nitrosoproline and its inhibition by Vitamin C. *Cancer Res.*, **46**, 1485.

MAGEE, P.N. (1989). The experimental basis for the role of nitrosocompounds in human cancer. *Cancer Surv.*, **8**, 207.

MARGISON, G.P., O'CONNOR, P.J., COOPER, D.P. & 7 others (1990). *$O^6$*-Alkylguanine DNA-alkyltransferase: significance, methods of measurement and some human tumour and normal tissue levels (Mini-workshop report). In *Triazenes: Chemical, Biological and Clinical aspects* Giraldi, T. & Connors, T. (eds). Plenum Press. pp. 195–206.

MYERS, K.A., SAFFHILL, R. & O'CONNOR, P.J. (1988). Repair of alkylated purines in the hepatic DNA of mitrochondria and nuclei in the rat. *Carcinogenesis*, **9**, 285.

O'CONNOR, P.J., FAN, C.Y., ZAIDI, S.M. & COOPER, D.P. (1991). Selective alkylation of cells in rat tissues after treatment with N-nitrosocompounds: immunohistochemical detection of potential target cells. In *Human Carcinogen Exposure: Biomonitoring and Risk Assessment*. Garner, R.C., Farmer, P.B., Steel, G.T. & Wright, A.S. (eds). Oxford University Press: Oxford (in press).

O'CONNOR, P.J., FIDA, S., BROMLEY, M. & SAFFHILL, R. (1988). Phenobarbital: a non-genotoxic agent which induces the repair of $O^6$-methylguanine from hepatic DNA. *Carcinogenesis*, **8**, 839.

O'CONNOR, P.J. & SAFFHILL, R. (1979). The action of rat cytosol enzymes on some methylated nucleic acid components produced by the carcinogenic *N*-nitrosocompounds. *Chem. Biol. Interact*, **26**, 91.

PEGG, A.E., (1983). Alkylation and subsequent repair of DNA after exposure to dimethylnitrosamine and related carcinogens. *Rev. Biochem. Toxicol.*, **5**, 83.

PEGG, A.E. & PERRY, W. (1981). Alkylation of nucleic acids and metabolism of small doses of dimethylnitrosamine in the rat. *Cancer Res.*, **41**, 3128.

PHILLIPS, D.H., HEWER, A., MARTIN, C.N., GARNER, R.C. & KING, M.M. (1988). Correlation of DNA adduct levels in the human lung with cigarette smoking. *Nature*, **336**, 790.

PRESTON-MARTIN, S. & CORREA, P.A. (1989). Epidemiological evidence for the role of nitrosocompounds in human cancer. *Cancer Surv.*, **8**, 459.

RANDERATH, E., MILLER, R.H., MITTAL, D., AVITTS, T.A., DUNSFORD, H.A. & RANDERATH, K. (1989). Covalent DNA damage in tissues of cigarette smokers as determined by $^{32}$P-postlabelling assay. *J. Natl Acad. Sci.*, **81**, 341.

SAFFHILL, R., BADAWI, A.F. & HALL, C.N. (1988*a*). The detection of $O^6$-methylguanine in human DNA. In *Methods for Detecting DNA Damaging Agents in Humans: Applications to Cancer Epidermiology and Prevention*. Bartsch, H., Hemminki, K. & O'Neill, I.K. (eds). Sci. Pub. No. 89 IARC Lyon, p. 301–305.

SAFFHILL, R., FIDA, S., BROMLEY, M. & O'CONNOR, P.J. (1988*b*). Promutagenic lesions are induced in the tissue DNA of animals treated with isoniazid. *Human Toxicol.*, **7**, 311.

SAFFHILL, R., MARGISON, G.P. & O'CONNOR, P.J. (1985). Mechanisms of carcinogenesis induced by alkylating agents. *Biochem. Biophys. Acta*, **823**, 111.

SCANLAN, R.A. (1983). Formation and occurrence of nitrosamines in food. *Cancer Res.*, **43**, (Suppl.) 2435.

SWENBERG, J.A., DRYOFF, M.C., BEDELL, M.A. & 4 others (1984). *$O^6$*-Ethyldeoxylthymidine but not *$O^6$*-ethyldeoxyguanosine, accumulates in hepatocyte DNA of rats exposed continuously to diethylnitrosamine. *Proc. Natl Acad. Sci. USA*, **81**, 1692.

UMBENHAUER, D., WILD, C.P., MONTESANO, R. & 7 others (1985). *$O^6$*-Methyldeoxyguanosine in oesophageal DNA among individuals at high risk of oesophageal cancer. *Int. J. Cancer*, **36**, 661.

WHO (1987). *Cancer Incidence in Five Continents* Vol V; Muir, C., Waterhouse, J., Mack, T., Powell, J. & Whelan, S. (eds). Sci. Pub. No. 88, IARC Lyon.

WIESTLER, O., KLEIHUES, P. & PEGG, A.E. (1984). $O^6$-Alkylguanine-DNA alkyltransferase activity in human brain and brain tumours. *Carcinogenesis*, **5**, 121.

WILD, C.P., MONTESANO, R., VAN BENTHEM, J., SCHERER, E. & DEN ENGELESE, L. (1990). Intercellular variation in levels of adducts of aflatoxin $B_1$ and $G_1$ in DNA from rat tissues: a quantitative immunocytochemical study. *J. Can. Res. Clin. Oncol.*, **116**, 134.

WILD, C.P., SMART, G., SAFFHILL, R. & BOYLE, J.M. (1983). Radioimmunoassay of $O^6$-methyldeoxyguanosine in the DNA cells alkylated *in vitro* and *in vivo*. *Carcinogenesis*, **4**, 1605.

WILSON, V.L., WESTON, A., MANCHESTER, D.K. & 8 others (1989). Alkyl and aryl carcinogen adducts detected in human peripheral lung. *Carcinogenesis*, **10**, 2149.

YAROSH, D.B. (1985). The role of $O^6$-methylguanine DNA methyltransferase in cell survival, mutagenesis and carcinogenesis. *Mutation Res.*, **145**, 1.

YOO, J.-S.H., GUENGERICH, P. & YANG, C.S. (1988). Metabolism of N-nitrosodialkylamines by human liver microsomes. *Cancer Res.*, **48**, 1499.